# Red Pine Bark Extract Alleviates Akt/GSK-3β Signaling Disruption in the Hippocampus of Streptozotocin-Induced Diabetic Sprague-Dawley Rats

**DOI:** 10.4014/jmb.2403.03038

**Published:** 2024-04-28

**Authors:** Kwan Joong Kim, Zukhra Akhmedova, Ho Jin Heo, Dae-Ok Kim

**Affiliations:** 1 Graduate School of Biotechnology, Kyung Hee University, Yongin 17104, Republic of Korea; 2 Division of Applied Life Science (BK21), Institute of Agriculture and Life Science, Gyeongsang National University, Jinju 52828, Republic of Korea; 3 Department of Food Science and Biotechnology, Kyung Hee University, Yongin 17104, Republic of Korea

**Keywords:** Apoptosis, diabetes mellitus, *Pinus densiflora*, tau protein

## Abstract

This study investigates whether red pine (*Pinus densiflora* Sieb. et Zucc.) bark extract (PBE) can alleviate diabetes and abnormal apoptosis signaling pathways in the hippocampus of streptozotocin (STZ)-induced diabetic Sprague-Dawley (SD) rats. Two dosages of PBE (15 and 30 mg/kg of body weight/day) were administered orally to STZ-induced diabetic SD rats for 20 days. Blood glucose level and body weight were measured once per week. After 20 days of oral administration of PBE, the rat hippocampus was collected, and the production of Akt, *p*-Akt, GSK-3β, *p*-GSK-3β, tau, *p*-tau, Bax, and Bcl-2 proteins were determined by western blot analysis. A decrease in blood glucose level and recovery of body weight were observed in PBE-treated diabetic rats. In the Akt/GSK-3β/tau signaling pathway, PBE inhibited diabetes-induced Akt inactivation, GSK-3β inactivation, and tau hyperphosphorylation. The protein production ratio of Bax/Bcl-2 was restored to the control group level. These results suggest that PBE, rich in phenolic compounds, can be used as a functional food ingredient to ameliorate neuronal apoptosis in diabetes mellitus.

## Introduction

Diabetes mellitus is a metabolic disorder that causes chronic hyperglycemia by destroying pancreatic beta cells and impairing insulin-mediated metabolism [1]. Because the brain relies on glucose as its primary source of energy, failure of insulin-mediated metabolism severely impairs central nervous system (CNS) function, impacting cognitive activity [2]. In particular, the hippocampus, which plays the most important role in forming long-term memories, is vulnerable to excitotoxicity caused by glucose depletion [2]. For this reason, research has focused on the relationship between diabetes mellitus and neurodegenerative diseases such as Alzheimer's disease (AD) and Parkinson's disease.

Although various factors contribute to the onset of neurodegenerative diseases accompanying diabetes mellitus, the phosphatidylinositol 3-kinase (PI3K)/protein kinase B (PKB; Akt) pathway is a major signaling pathway [3]. Activation of the PI3K/Akt pathway promotes cell survival, including neurons in the CNS, and regulates glucose metabolism [3]. However, when insulin-mediated metabolism is damaged due to diabetes, PI3K/Akt activation may be inhibited, resulting in neuronal damage [3]. In PI3K/Akt-inactivated metabolism, apoptotic regulator proteins such as Bax and Bcl-2 may become unbalanced and activate caspase-3, leading to neuronal apoptosis [4]. In addition, PI3K/Akt inactivation induces the activation of glycogen synthase kinase-3 β (GSK-3β), which leads to tau hyperphosphorylation, one of the most potent causes of AD [5, 6].

Recent studies have reported that plant-derived phenolic compounds upregulate the PI3K/Akt pathway, which is damaged in diabetes mellitus [5-7]. It was previously reported that polyphenols including catechins and procyanidins in lychee seed inhibited tau hyperphosphorylation by upregulating protein expression of insulin receptor substrate-1/PI3K/Akt and downregulation of GSK-3β [6]. Quercetin, a plant-derived polyphenol, has been reported to have neuroprotective effects by activating Akt and reducing cleaved caspase-3 protein in rats with focal cerebral ischemia [8]. These results suggest that plant-derived phenolic compounds may be potent neuroprotective agents against diabetes mellitus-induced neurodegenerative diseases.

The bark of *Pinus densiflora* Sieb. et Zucc. (red pine), which is discarded as a by-product of the wood industry, contains various phenolic compounds such as taxifolin, catechin, and procyanidins [9-12]. Red pine bark extract has been reported to have antidiabetic, antioxidant, antihypertensive, and neuroprotective effects [9, 10, 12] and can be used as a high-value functional food and pharmaceutical material. A previous study reported evidence for the neuroprotective action of catechin, procyanidins, and taxifolin, the major phytochemicals in red pine bark [12], but no studies have examined the effects of red pine bark in animal models of diabetes mellitus. Therefore, in this study, we confirmed the anti-apoptotic effect of red pine bark by investigating the Akt/GSK-3β pathway and tau protein expression levels in the hippocampus of Sprague-Dawley (SD) rats with diabetes induced by streptozotocin (STZ).

## Materials and Methods

### Chemicals

Hot water-extracted red pine bark extract (PBE; trade name PineXol) was supplied by Nutrapharm Co., Ltd.(Korea). STZ was purchased from Alfa Aesar (USA). Lysis buffer was obtained from Noble Bio, Inc. (Korea), and protease inhibitor cocktail and phosphatase inhibitor cocktail were purchased from GenDEPOT (USA). Primary antibodies Bcl-2, Bax, Akt, *p*-Akt (serine [Ser]473), GSK-3β, *p*-GSK-3β (Ser9), tau, *p*-tau (Ser396 and Ser404), and GAPDH; and horseradish peroxidase (HRP)-conjugated anti-mouse and anti-rabbit (secondary antibody) were purchased from Abcam plc (UK). Bovine serum albumin (BSA), sodium dodecyl sulfate (SDS), ammonium persulfate, skim milk powder, and polysorbate 20 (Tween 20) were purchased from Sigma-Aldrich, Inc. (USA).

### Animals

Twenty-four male SD rats, five weeks old and weighing approximately 200 g, were purchased from Saeron Bio Inc. (Korea). Rats were housed in laboratory cages controlled at a temperature of 23°C, relative humidity of 56%, and 12 h light/dark cycle from 8:00 a.m. to 8:00 p.m. Rats had access to standard chow diet (5L79; Orient Bio Inc., Korea) and water *ad libitum* throughout the experimental period.

### Oral Administration of PBE

**Fig. 1:**
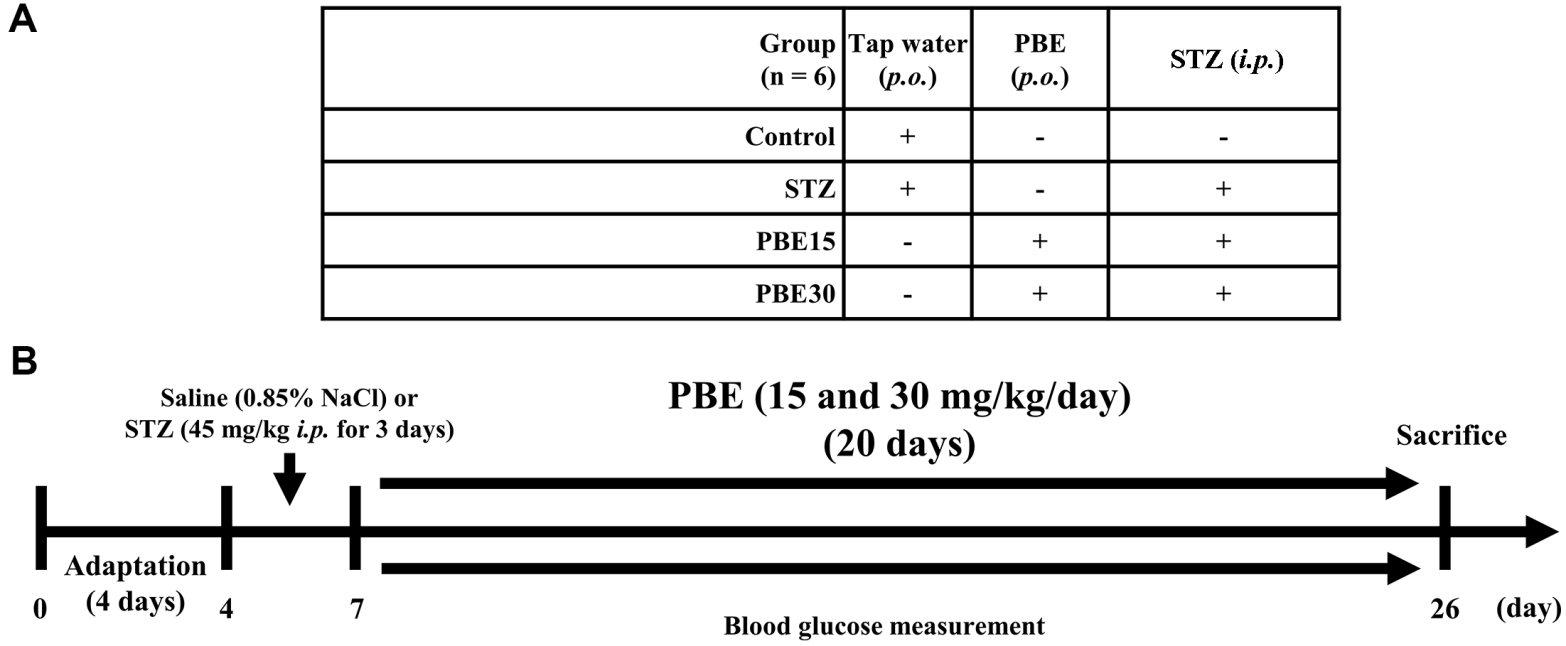
(A) Experimental group information and (B) timeline of animal experiments. PBE15 group, oral administration of 15 mg of PBE/kg of BW/day to SD rats; PBE30 group, oral administration of 30 mg of PBE/kg of BW/day to SD rats; BW, body weight; *i.p.*, intraperitoneal injection; PBE, pine (*Pinus densiflora* Sieb. et Zucc.) bark extract; *p.o., per os*; SD, Sprague-Dawley; STZ, streptozotocin.

The rats were randomly assigned to the control group (6 rats) or the STZ-treatment group (18 rats). After a four-day adaptation period (onset of study), 1 ml of saline (0.85% NaCl) and STZ (45 mg/kg of body weight [BW]) in 1 ml saline were intraperitoneally injected in control rats (control group) and STZ-induced rats for three days, respectively (Fig. 1). Three days after intraperitoneal injections were complete, the 18 STZ-induced rats were randomly assigned to one negative control group (STZ group; *n* = 6) and two treatment groups (PBE15 and PBE30 groups; *n* = 6, respectively). Then, tap water (control and STZ groups), 15 mg of PBE/kg of BW/day (PBE15 group), or 30 mg of PBE/kg of BW/day (PBE30 group) in tap water were orally administered to rats at 9 a.m. each day for 20 days.

### Measurement of Fasting Blood Glucose Level

After fasting for 12 h, blood glucose was measured by collecting blood from the tail vein once per week for 20 days using a glucose meter (ACCU-CHEK; Roche Diagnostics, Germany). The first and last measurements of fasting blood glucose were made on days 1 and 20 of oral administration.

### Western Blot Analysis

After administering PBE for 20 days, the animals were anesthetized with isoflurane, and the hippocampus was quickly harvested. Hippocampi were homogenized in lysis buffer. Homogenized tissue in lysis buffer was sonicated (NRE-02; Next Advance, USA) and centrifuged at 18,403 ×*g* for 20 min at 4°C using a centrifuge (PK121R; ALC International Srl, Italy). Supernatants of the tissue lysates were collected and stored at -80°C prior to analysis.

Protein concentration was quantified by Bradford protein assay. Samples were separated using 10% SDS-PAGE, transferred to polyvinylidene fluoride membranes, and blocked with 5% skim milk or 5% BSA dissolved in Tris-buffered saline containing 0.1% Tween 20 (TBST) for 1 h at room temperature. The membrane was incubated with primary antibodies (rabbit polyclonal to Akt [ab8805], dilution 1:1000; rabbit monoclonal to *p*-Akt [ab81283], dilution 1:1000; rabbit polyclonal to GSK-3β [ab131356], dilution 1:1000; rabbit polyclonal to *p*-GSK-3β [ab107166], dilution 1:1000; rabbit polyclonal to tau [ab64193], dilution 1:1000; rabbit monoclonal to *p*-tau (Ser396) [ab109390], dilution 1:1000; rabbit monoclonal to *p*-tau (Ser404) [ab92676], dilution 1:1000; rabbit polyclonal to Bcl-2 [ab59348], dilution 1:1000; rabbit polyclonal to Bax [ab53154], dilution 1:1000, and mouse monoclonal to GAPDH [ab9484], dilution 1:1000) overnight at 4°C with agitation, washed three times with TBST, and then blotted with secondary antibodies (1:2500 dilution) for 1 h at room temperature. After reactions, the membrane was visualized with enhanced chemiluminescence solution (EzWestLumi plus; ATTO, USA) reacting with HRP and analyzed using ImageJ software (version 1.49; USA).

### Statistical Analysis

All data are expressed as the mean ± standard deviation. Statistical analysis was performed using SPSS software (version 23.0; IBM SPSS Statistics Inc., USA). The significance of differences of average values was analyzed through one-way analysis of variance, followed by Tukey’s multiple comparison test (*p* < 0.05). Significance is indicated by asterisk and hash.

## Results

### Effects of PBE on BW and Fasting Blood Glucose of STZ-Induced Diabetic SD Rats

BW and blood glucose measurements of SD rats on days 4, 7, and 26 of the experimental period are displayed in Table 1. We observed no significant differences in the BW of rats before saline or STZ injection in any groups (Table 1). Three days after intraperitoneal injections of saline or STZ, there was no significant difference in BW among groups as before intraperitoneal injection (day 4). However, at the end of the experiment (day 26), the control, PBE15, and PBE30 groups all had significantly higher BW compared to the STZ group.

There were no significant differences in the fasting blood glucose levels of rats in any groups before saline or STZ injection (day 4). Three days after intraperitoneal injection of saline or STZ into rats, the blood glucose levels of the STZ-injected rats (STZ, PBE15, and PBE30 groups) were increased to an average of 407.8 mg/dl compared to the control rats (average 117.1 mg/dl) (Table 1). At the end of the experiment (day 26), the PBE15 and PBE30 groups showed 23.0% and 20.2% decreases in blood glucose levels respectively, compared to the STZ group (Table 1).

### Effects of PBE on Apoptosis-Related Protein Production in the Hippocampus of STZ-Induced Diabetic SD Rats

**Fig. 2:**
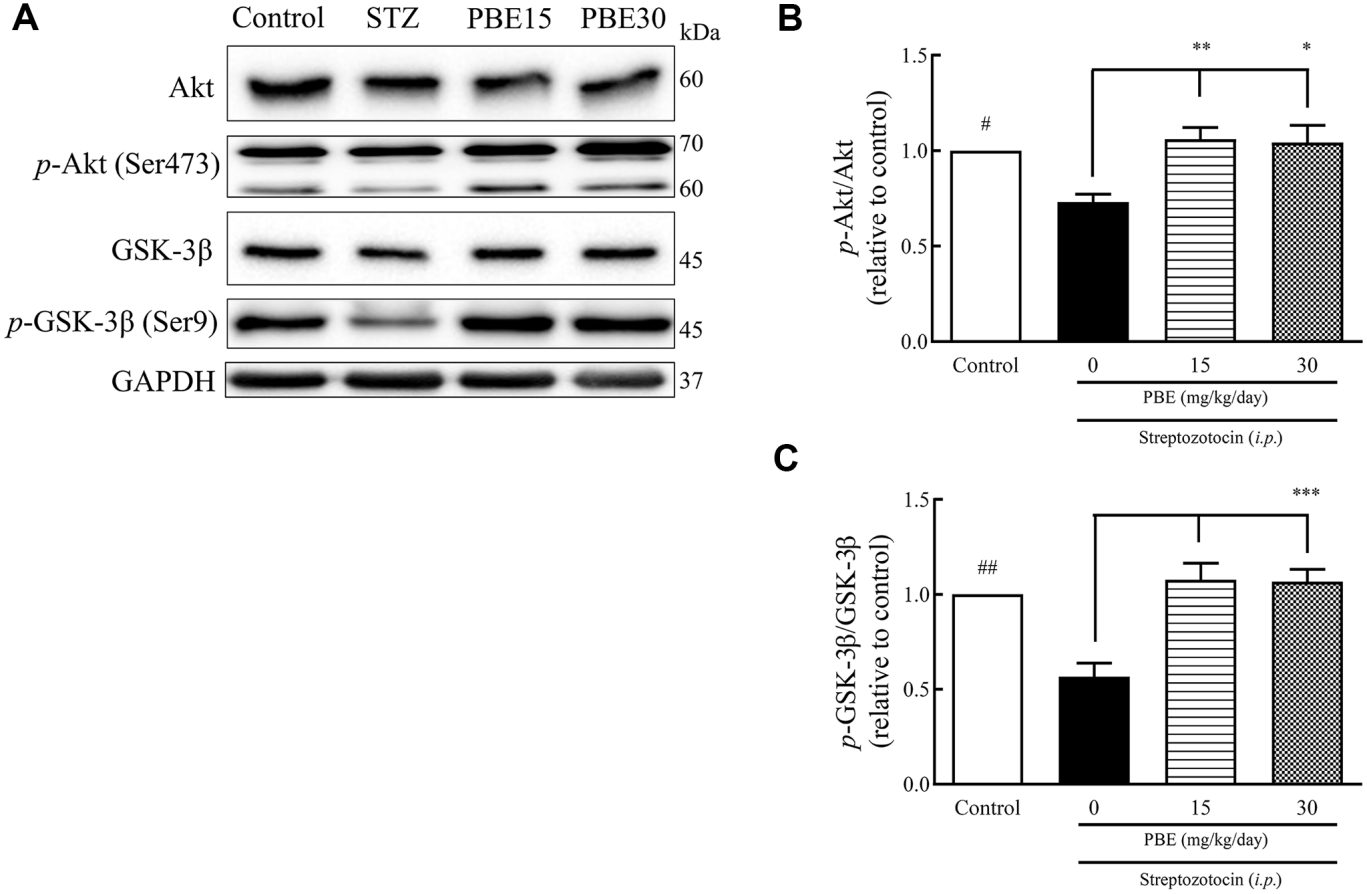
Effects of PBE on Akt, *p*-Akt, GSK-3β, and *p*-GSK-3β production in the hippocampus of STZ-induced diabetic Sprague-Dawley rats. Representative western blot, *p*-Akt/Akt ratio, and *p*-GSK-3β/GSK-3β ratio. Protein production was determined using western blot analysis. STZ (45 mg/kg of BW) dissolved in 1 ml saline was injected intraperitoneally into STZ-induced rats for three days. Significant differences by Tukey’s multiple comparison test: ^#^*p* < 0.05 and ^##^*p* < 0.01 vs. control, *p < 0.05, **p < 0.01, and ***p < 0.001 vs. STZ. BW, body weight; PBE, pine (*Pinus densiflora* Sieb. et Zucc.) bark extract; STZ, streptozotocin.

After 20 days of treatment, the production of proteins related to neuronal apoptosis in the rat hippocampus was measured through western blot analysis (Fig. 2). The production of Akt, *p*-Akt (Ser473), GSK-3β, and *p*-GSK-3β (Ser9) in the hippocampus are shown as western blot bands (Fig. 2A). The *p*-Akt/Akt ratios of the STZ, PBE15, and PBE30 groups were 0.73, 1.06, and 1.04, respectively (Fig. 2B). The *p*-Akt/Akt ratio decreased by 23.0% in the STZ group compared to the control group (Fig. 2B). The PBE15 and PBE30 groups showed 45.2% and 42.5% increases in the *p*-Akt/Akt ratio, respectively, compared to the STZ group (Fig. 2B). The *p*-GSK-3β/GSK-3β ratios of the STZ, PBE15, and PBE30 groups were 0.57, 1.08, and 1.07, respectively (Fig. 2C). The *p*-GSK-3β/GSK-3β ratio decreased by 43.4% in the STZ group compared to the control group (Fig. 2C). The PBE15 and PBE30 groups showed an 89.5% and 87.7% increase in the *p*-GSK-3β/GSK-3β ratio, respectively, compared to the STZ group (Fig. 2C).

**Fig. 3:**
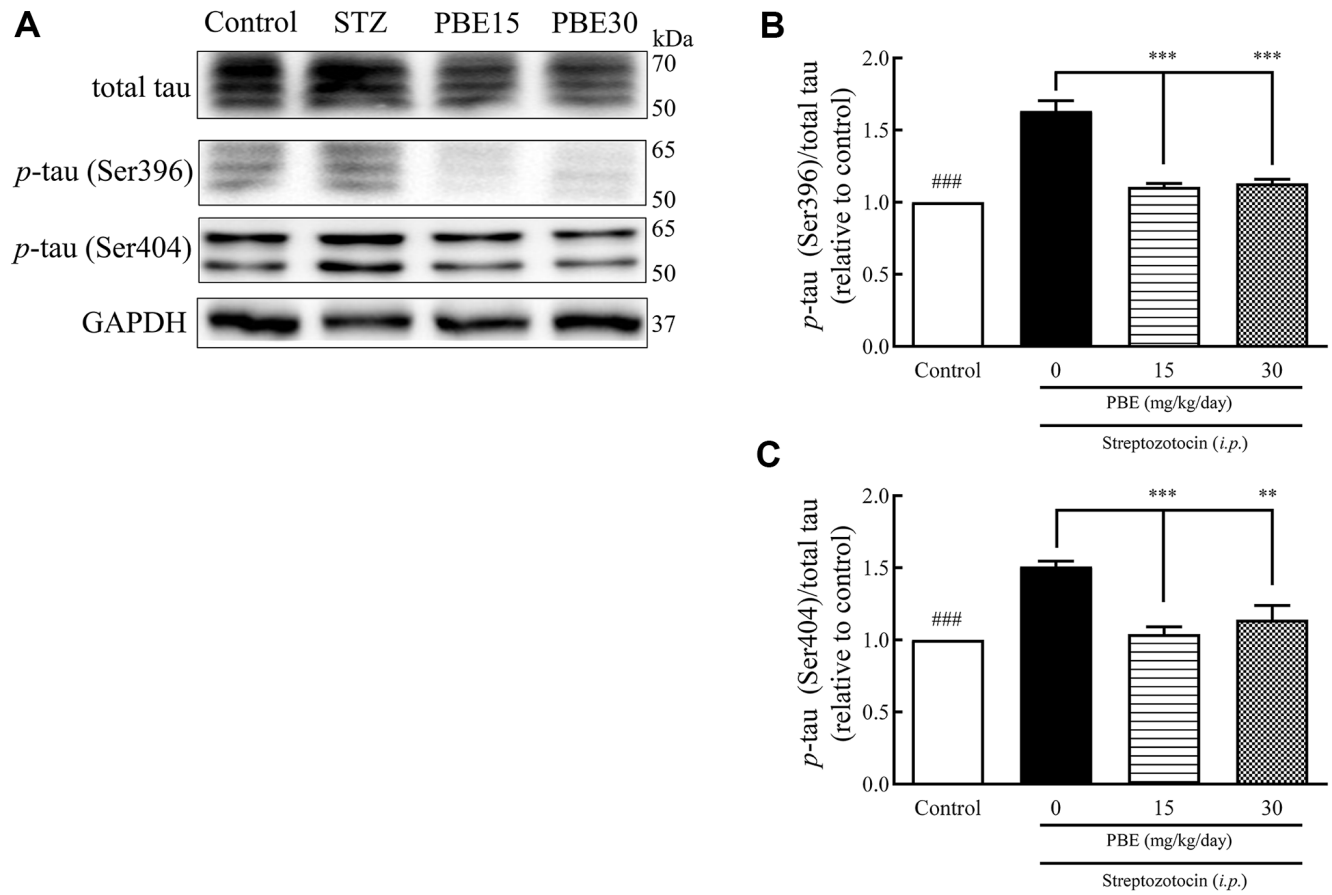
Effects of PBE on total tau and *p*-tau (Ser396 and Ser404) production in the hippocampus of STZinduced diabetic Sprague-Dawley rats. Representative western blot, *p*-tau (Ser396)/total tau ratio, and *p*-tau (Ser404)/total tau ratio. Protein production was determined by western blotting. STZ (45 mg/kg of BW) dissolved in 1 ml saline was injected intraperitoneally into STZ-induced rats for three days. Significant differences by Tukey’s multiple comparison test: ^###^*p* < 0.001 vs. control, and **p < 0.01 and ***p < 0.001 vs. STZ. BW, body weight; PBE, pine (*Pinus densiflora* Sieb. et Zucc.) bark extract; STZ, streptozotocin.

The production of total tau and *p*-tau (Ser396 and Ser404) in the hippocampus is shown as western blot bands (Fig. 3A). The *p*-tau (Ser396)/total tau ratios of the STZ, PBE15, and PBE30 groups were 1.63, 1.11, and 1.13, respectively (Fig. 3B), with an increase of approximately 63.0% in the STZ group compared to the control group (Fig. 3B). The *p*-tau (Ser396)/total tau ratio of the PBE15 and PBE30 groups decreased by approximately 31.9%and 30.7%, respectively, compared to the STZ group (Fig. 3B). The *p*-tau (Ser404)/total tau ratios of the STZ, PBE15, and PBE30 groups were 1.51, 1.04, and 1.14, respectively (Fig. 3C), increasing by approximately 51.0% in the STZ group compared to the control group (Fig. 3C). The *p*-tau (Ser404)/total tau ratios of the PBE15 and PBE30 groups decreased by approximately 31.1% and 24.5%, respectively, compared to the STZ group (Fig. 3C).

**Fig. 4:**
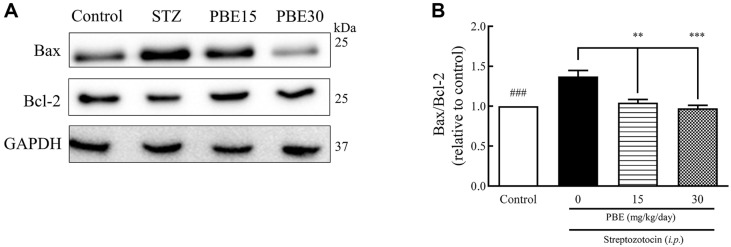
Effects of PBE on Bax and Bcl-2 production in the hippocampus of STZ-induced diabetic Sprague-Dawley rats. Representative western blot and Bax/Bcl-2 ratio. Protein production was determined by western blotting. STZ (45 mg/kg of BW) dissolved in 1 ml saline was injected intraperitoneally into STZ-induced rats for three days. Significant differences by Tukey’s multiple comparison test: ^###^*p* < 0.001 vs. control, **p < 0.01 and ***p < 0.001 vs. STZ. BW, body weight; PBE, pine (*Pinus densiflora* Sieb. et Zucc.) bark extract; STZ, streptozotocin.

The western blot bands of Bax and Bcl-2 production in the hippocampus are shown in Fig. 4A. The Bax/Bcl-2 ratios of the STZ, PBE15, and PBE30 groups were 1.37, 1.05, and 0.97, respectively (Fig. 4B), with a 34.0% increase in Bax/Bcl-2 ratio in the STZ group compared to the control group (Fig. 4B). The PBE15 and PBE30 groups showed a 23.3% and 29.2% decrease in the Bax/Bcl-2 ratio, respectively, compared to the STZ group (Fig. 4B).

## Discussion

When STZ is metabolized, it is absorbed into insulin-producing cells, such as pancreatic b-cells, and affects alkylation of the b-cell DNA by producing reactive oxygen/nitrogen species, such as superoxide, hydrogen peroxide, and nitric oxide [13-15]. Excessive oxidative stress in the pancreas interferes with insulin synthesis and causes diabetes mellitus with hyperglycemia, which leads to many complications in the body [16, 17]. In particular, high glucose level in the hippocampus, which plays an important role in long-term memory, cause cognitive impairment due to neuronal apoptosis [18]. Apoptosis in the diabetic hippocampus is mainly affected by the PI3K/Akt signaling pathway, which regulates apoptosis by insulin metabolism [3]. An impaired PI3K/Akt signaling pathway inhibits Akt activation and induces GSK activation, which increases tau phosphorylation and forms neurofibrillary tangles [5, 6, 19, 20]. Neurofibrillary tangles create excessive oxidative stress in the brain or hippocampus and induce neuronal apoptosis [19]. In the present study, to evaluate whether PBE can alleviate the abnormal neuronal apoptosis signaling pathway caused by diabetes in the hippocampus, STZ was intraperitoneally administered to SD rats to induce diabetes. The expressions of Akt and GSK-3β, a key signaling pathway for glucose metabolism, tau phosphorylation, and apoptosis regulator proteins Bax and Bcl-2, were measured.

Typical phenotypes of STZ-induced diabetic rats are increased blood glucose levels, weight loss, frequent water intake due to thirst, and polyuria [21]. Three days after intraperitoneal administration of STZ in this study, the diabetic rats showed higher water intake and short straw litter replacement cycles compared to the control group. Another typical symptom of diabetes was an average blood glucose level of 350 mg/dl, with some rats showing levels up to 500 mg/dl, which is considered to represent severe hyperglycemia. Additionally, diabetic rats showed about 40% lower body weight than the control group at the beginning and end of the experiment. These results indicate that diabetes was successfully induced in rats by intraperitoneal administration of STZ.

Plant-based extracts have been widely studied and used to alleviate diabetes mellitus due to their rich antioxidant phenolic compounds [6, 22]. Pine bark, including *P. densiflora*, has been reported to have α-glucosidase inhibitory activity to regulate blood glucose levels [23]. In addition, it was previously reported that extract and fraction of *P. densiflora* bark and its main phenolic compounds exert antidiabetic effects by upregulating b-cell insulin secretion and increasing cell uptake in rat skeletal muscle cells [24]. In the present study, diabetic rats administered PBE tended to have decreased blood sugar levels and increased body weight compared to diabetic rats at the end of the experiment. These results suggest that PBE can alleviate STZ-induced diabetes.

Intraperitoneally administered STZ is metabolized and induces pancreatic b-cell disruption, resulting in abnormal functioning of insulin signaling and secretion. Primarily, the insulin receptor substrate is unable to transmit signals from the insulin and insulin-like growth factor-1 receptors to the cellular PI3K/Akt pathway, inhibiting Akt phosphorylation at Ser473, affecting apoptotic regulator proteins such as Bax and Bcl-2, and negatively regulating cellular survival and growth [3, 25]. Additionally, inactivated Akt directly upregulates the phosphorylation of GSK-3β at Ser9 to inhibit glycogen synthase and induces its pro-apoptotic activity [3, 5]. Activated GSK-3β has been reported to decrease long-term memory formation and cause cognitive impairment in the hippocampus [18, 20, 26]. Previously, phenolic compounds from lychee seed, including catechins and procyanidins, were reported to improve the function of the IRS-1/PI3K/Akt/GSK-3β pathway and downregulate AD-associated tau expression in insulin-resistant cells [6]. Procyanidins, one of the main groups of bioactive compounds in PBE, have been reported to ameliorate PI3K/Akt signaling pathway perturbation caused by high glucose in ganglion neurons [7]. In the current study, the STZ-induced diabetic group exhibited downregulated expressions of *p*-Akt (Ser473) and *p*-GSK-3β (Ser9) compared to the control group, while their expressions were upregulated in the hippocampus of PBE-treated diabetic rats. Considering that PBE contains several phenolic compounds, including procyanidins and catechins, our results are similar to previous findings and suggest that PBE can improve the dysfunction of the PI3K/Akt/GSK-3β signaling pathway induced by STZ.

Tau protein is abundant in the CNS neurons, typically in the axons, which conduct neurotransmission between neurons. Hyperphosphorylation of tau protein promotes aggregation into paired helical filaments, forms neurofibrillary tangles (NFTs), and induces synaptic disconnection [27]. NFTs are a potent cause of AD because they induce excessive oxidative stress in neurons and lead to neuronal apoptosis [19]. GSK-3β promotes hyperphosphorylation of tau protein, leading to NFT formation and neuronal apoptosis [27]. For these reasons, many studies have focused on the phytotherapeutic effects of antioxidant phenolic compounds that regulate the Akt/GSK-3β/tau pathway and improve neurodegenerative diseases such as AD [5, 6]. In our study, PBE reduced diabetes-induced increased tau phosphorylation by regulating Akt/GSK-3β, which is consistent with previous studies [5, 6].

Bax is a pro-apoptotic protein, while Bcl-2 is an anti-apoptotic protein, so the ratio of the these two proteins determines the apoptotic or anti-apoptotic state of the cell. Administration of STZ increases Bax production and decreases Bcl-2 production, resulting in upregulation of apoptotic signaling activities, such as caspase-3 [3]. The Bax/Bcl-2 ratio was increased in the hippocampus of the STZ-induced diabetic group but restored in the PBE-treated groups. The lower Bax/Bcl-2 ratio in the PBE-treated group suggests that hippocampal neurons are better protected against apoptosis compared to the STZ-induced diabetic group. However, the specific mechanisms responsible for these changes are not entirely clear, as various factors, such as modulation of the Akt/GSK-3β/tau pathway and radical scavenging by PBE, may influence the action of apoptosis regulators.

PBE prevented neuronal death in STZ-induced diabetic rats by regulating Akt/GSK-3β, tau hyperphosphorylation, and Bax/Bcl-2 protein. There is evidence of the antidiabetic effect of PBE, as seen in previous reports on the antidiabetic effect of *P. densiflora* and the phytochemical constitution of PBE [12, 28]. However, the effects of PBE on diabetes-induced hippocampus damage have not been explored. Our findings that PBE regulates several crucial indicators related to neurotransmission, including Akt, GSK-3β, tau, and Bax/Bcl-2 in the hippocampus in diabetic rats, suggest a potential health-beneficial effect of PBE. However, our results have limitations for explaining the antidiabetic effects of PBE. We did not conduct glucose tolerance test, the classic test used to diagnose diabetes, and only some of the PI3K/Akt signaling pathways (Akt/GSK-3β) of diabetes were evaluated. Therefore, further research is needed to fully understand the antidiabetic effects of PBE. In summary, this study provides evidence for the protective effects of PBE against diabetes-induced hippocampal dysfunctions and supports the potential application of PBE in pharmaceuticals or nutraceuticals.

## Figures and Tables

**Table 1:** Effects of red pine (*Pinus densiflora* Sieb. et Zucc.) bark extract (PBE) on body weight (BW) and fasting blood glucose levels of streptozotocin (STZ)-induced diabetic Sprague-Dawley (SD) rats.

Group	Body weight (g)				Plasma glucose level (mg/dl)		
	Onset of study (day 4)	Three days after STZ injection (day 7)	End of study (day 26)		Onset of study (day 4)	Three days after STZ injection (day 7)	End of study (day 26)
Control	140.7 ± 3.3	227.1 ± 5.1	268.4 ± 5.1		120.5 ± 4.0	115.6 ± 2.1	117.5 ± 2.7
STZ	141.5 ± 1.9	204.3 ± 5.6	212.9 ± 6.3^###^		120.8 ± 3.8	408.4 ± 61.9^###^	445.5 ± 64.2^###^
PBE15	140.1 ± 3.9	215.4 ± 5.0	254.4 ± 12.8*		115.1 ± 3.5	407.5 ± 51.6	343.0 ± 56.2***
PBE30	140.9 ± 2.4	206.6 ± 3.9	255.2 ± 10.2*		115.1 ± 2.7	407.4 ± 58.6	355.6 ± 65.9***

Data are expressed as mean ± standard deviation (*n* = 6). Significant differences by Tukey’s multiple comparison test: ^###^*p* < 0.001 vs. control, *p < 0.05 and ***p < 0.01 vs. STZ. PBE15 group, oral administration of 15 mg of PBE/kg of BW/day to SD rats; PBE30 group, oral administration of 30 mg of PBE/kg of BW/day to SD rats.
